# Specific Collagen Peptides Support Postprandial Metabolic Health by Modulating Incretin Hormones, Gastric Transit, and Glycemic Control in Participants with Normoglycemia and Prediabetes

**DOI:** 10.1016/j.cdnut.2026.109512

**Published:** 2026-08-08

**Authors:** Nicolina Virgilio, Christiane Schön, Nicole Steiner, Vincent Schlageter, Manfred Wilhelm, Catarina IF Silva, Elien Gevaert, Janne Prawitt

**Affiliations:** 1 Department of Innovation, Rousselot BV, Ghent, Belgium; 2 Nutritional CRO, BioTeSys GmbH, Esslingen, Germany; 3 R&D, Motilis Medica SA, Lausanne, Switzerland; 4 Department of Mathematics, Natural and Economic Sciences, Ulm University of Applied Sciences, Ulm, Germany

**Keywords:** GLP-1, glucose management, insulin response, incretin response, collagen peptides, gastric transit, nutritional strategy

## Abstract

**Background:**

Nutritional preload strategies can improve postprandial glycemic control through modulation of incretin secretion. We previously identified specific collagen peptides (CP; Nextida GC) that stimulate glucagon-like peptide-1 (GLP-1) secretion in vitro and improve postprandial glycemic responses in preclinical models and a pilot human study.

**Objectives:**

This study aims to investigate the effects of a CP preload on postprandial glycemic control and to characterize the associated hormonal and physiological responses in participants with normoglycemia or prediabetes.

**Methods:**

In a randomized, double-blind, placebo-controlled, crossover study, 30 participants (12 normoglycemic and 18 prediabetic) ingested 10 g CP or placebo 30 min before a standardized carbohydrate-rich meal (preload phase). Plasma glucose, serum insulin, C-peptide, GLP-1, and glucose-dependent insulinotropic polypeptide (GIP) concentrations were measured during the preload and postprandial periods. As a potential physiological mechanism, gastric transit was assessed via the gastric residence time of an indigestible capsule.

**Results:**

During the preload phase, CP significantly increased insulin and incretin levels (GLP-1 and GIP) compared with placebo (all *P* < 0.0001). After the meal, CP significantly reduced postprandial glucose excursions (incremental area under the curve (iAUC)_0-180 min_, *P* = 0.0178), whereas total insulin responses were unchanged (iAUC_-30-180 min_, *P* = 0.8929). Furthermore, CP prolonged the gastric residence time of the indigestible 3D-Transit capsule in the prediabetic subgroup (*P* = 0.0436).

**Conclusions:**

A 10 g preload of CP improved postprandial glycemic control without increasing overall postprandial insulin exposure. These effects were accompanied by early stimulation of insulin and incretin hormones and, in individuals with prediabetes, prolonged gastric residence time. The findings suggest that CP may support healthy postprandial metabolic regulation through complementary hormonal and physiological mechanisms.

This study was registered at clinicaltrials.gov as NCT06789263.

## Graphical abstract

**Figure:**
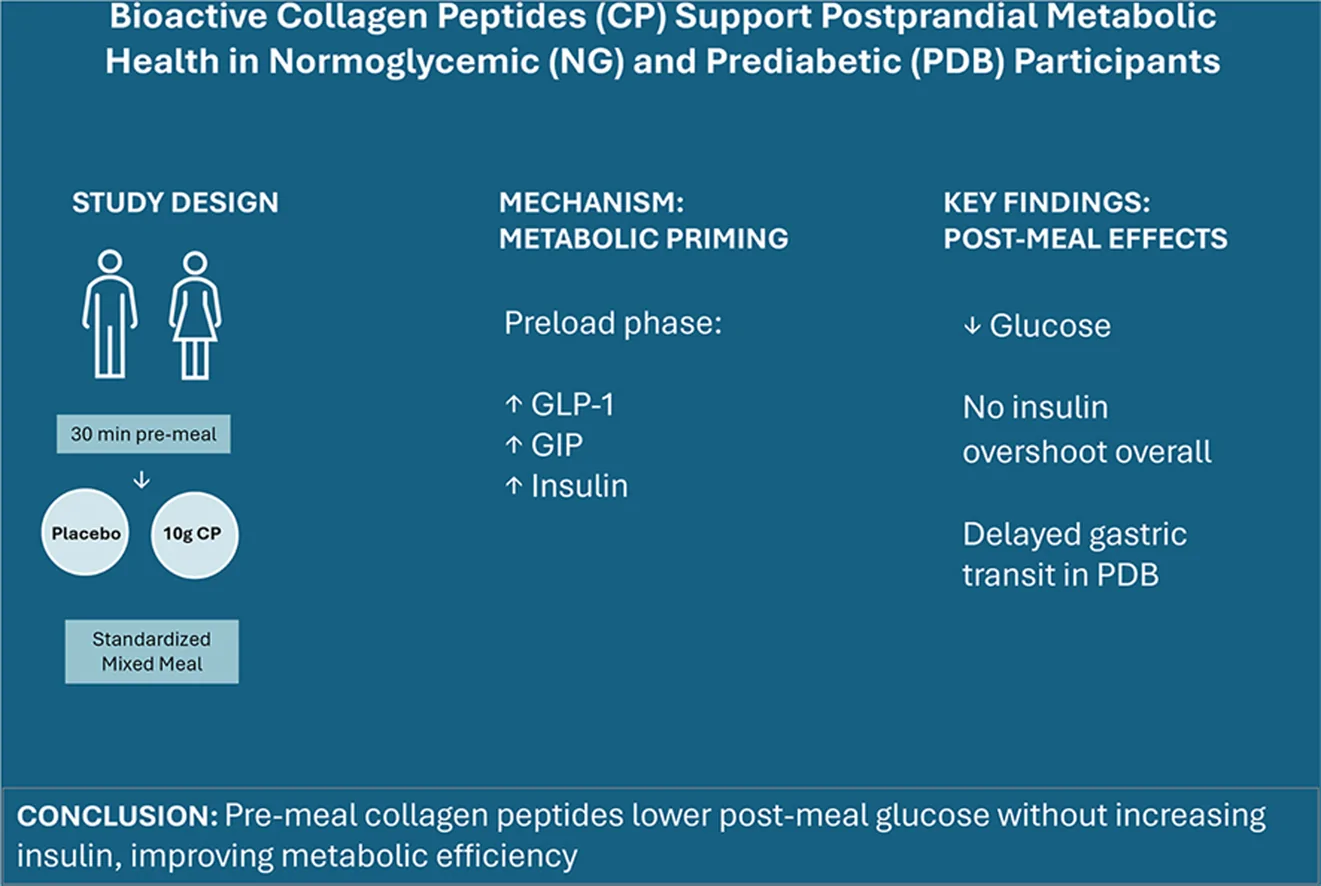

## Introduction

Maintaining optimal postprandial glucose and insulin responses is fundamental to metabolic health. Dysregulation of these processes can contribute to the development of prediabetes, type 2 diabetes, and cardiovascular disease [1,2]. In addition, elevated glycemic variability has been associated with adverse functional outcomes, including mood disturbances, increased food cravings, and appetite dysregulation, which may further reinforce unhealthy eating behaviors, creating a vicious cycle [3,4]. Consequently, strategies that improve postprandial glycemic control and reduce excessive insulin secretion are of considerable clinical and nutritional interest [5,6].

Glycemia is among the most tightly regulated physiological parameters in humans. The incretin hormones glucagon-like peptide-1 (GLP-1) and glucose-dependent insulinotropic polypeptide (GIP), secreted by intestinal cells after food intake, are key regulators of postprandial metabolism [7]. They enhance insulin secretion and regulate gastric emptying [8], thereby influencing the rate of nutrient delivery and absorption [[9], [10], [11]]. Their actions are highly interconnected: GLP-1 can slow gastric emptying and thereby indirectly reduce GIP release, while also exerting direct inhibitory effects on GIP activity. Conversely, GLP-1 and GIP may act synergistically to enhance insulin secretion [12,13].

Gastric emptying is a key determinant of postprandial glycemia, accounting for ∼35% of the variability in peak glucose levels [14]. By controlling the rate at which nutrients enter the small intestine [15], gastric emptying plays a central role in healthy individuals in regulating glucose absorption and maintaining subsequent healthy metabolic responses.

Nutritional preload strategies, consuming a small amount of macronutrients before a meal, have been shown to attenuate postprandial glycemic excursions through incretin stimulation and delayed gastric emptying. Whey protein is currently considered a benchmark preload intervention. However, its efficacy typically requires relatively high doses (15–50 g) and is often accompanied by a pronounced insulin response driven by branched-chain amino acids [16].

Collagen peptides (CP) are a complex mixture of oligopeptides with a common mean molecular weight between 1 and 5 kDa, which are derived from enzymatic hydrolysis of denatured collagen polypeptides (∼100 kDa). Tailoring parameters of the enzymatic hydrolysis process allows generating CPs with distinct compositional and functional properties. Although a limited number of reports suggest that certain CPs may modulate glucose control [17,18], we have recently identified specific CPs (Nextida GC) with enhanced GLP-1 secretory activity in vitro. Preclinical evidence suggests that CP may improve glycemic regulation through enhanced incretin release and potentially through delayed gastric emptying. In an exploratory pilot study, CP reduced postprandial glycemic responses in humans [19].

Despite these promising findings, the effects of CP on the integrated regulation of postprandial metabolism in humans remain poorly characterized. Moreover, it is unclear whether these effects differ according to the metabolic phenotype. We hypothesized that a CP preload would stimulate endogenous GLP-1 secretion in humans and improve postprandial glycemic responses while limiting the need for a compensatory increase in insulin secretion.

Therefore, the aim of this study was to determine whether a 10-g CP preload improves postprandial glycemic control through the modulation of incretin responses and gastric transit and to evaluate whether these effects differ between normoglycemic and prediabetic individuals.

## Methods

### Study design

This study was conducted as a randomized, double-blind, placebo-controlled, 2-way crossover study with a washout period of 14 ± 7 d between interventions at BioTeSys GmbH. The protocol and the informed consent form were approved by the Institutional Review Board of the Landesärztekammer Baden-Württemberg (F-2024-114) and registered at clinicaltrials.gov (NCT06789263). The study was performed in accordance with the Declaration of Helsinki and the principles of the International Council for Harmonisation Good Clinical Practice. Participants were recruited between January and September 2025, and all gave written informed consent.

Men and women aged 18–70 y with a BMI of 19–35 kg/m^2^ were eligible for participation. Participants were required to be either normoglycemic, defined as fasting glucose <100 mg/dL and hemoglobin A1c (HbA1c) <5.7%, or prediabetic, defined as HbA1c 5.7%–6.4% and/or fasting plasma glucose 100–125 mg/dL. In cases with HbA1c <5.7%, elevated fasting glucose had to be confirmed on 2 separate days. Additional inclusion criteria were nonsmoking status, stable dietary habits and physical activity during the 3 months before screening and throughout the study, and stable chronic medication, if applicable, for ≥4 wk before screening.

Main exclusion criteria were diagnosed type 2 diabetes requiring medical treatment; diseases or medications known to affect digestion, nutrient absorption, or glucose metabolism; severe hepatic or renal disease; clinically relevant gastrointestinal inflammatory or malignant disease; pregnancy or lactation; substance abuse; recent major illness or surgery; marked recent weight change; use of supplements known to influence glucose tolerance; recent blood donation; participation in another clinical study; or any condition that could impair safety or compliance in the investigator’s judgment.

### Study products and interventions

The investigational product consisted of CPs produced through enzymatic hydrolysis of porcine skin gelatin following a patented manufacturing process (Nextida GC, Rousselot, BV). The product had an average molecular weight of 3.5 kDa and was functionally tested for its capacity to enhance GLP-1 secretion in vitro. The product had a greater GLP-1 secretory activity compared with CPs generated using alternative enzymatic hydrolysis processes [19]. Blinding was ensured by flavoring both the study product and the placebo (water) with a cherry-flavored maltodextrin base (1 g maltodextrin per serving) (FOODAROM). This flavor profile was selected to mask potential taste or mouthfeel differences of the 10 g peptide load, with sensory equivalence and blinding efficacy prevalidated through internal tasting sessions by a trained sensory panel.

The randomization list was generated using RandList (DatInf GmbH). Study products were labeled with the randomization number and visit number. After an overnight fast of ≥10 h, participants consumed a single dose of either placebo or 10 g CP dissolved in 200 mL of water 30 min before the mixed meal. The mixed meal, standardized to 75 g of carbohydrates, consisted of white bread with butter and strawberry jam and was served at time 0 min. The meal provided 533 kcal (fat: 20.9 g, carbohydrates: 75 g, protein: 9.2 g). The intake of 10 g CP 30 min before the mixed meal contributed ∼41 kcal, corresponding to an increase in total energy content of 7.69%.

**FIGURE 1:**
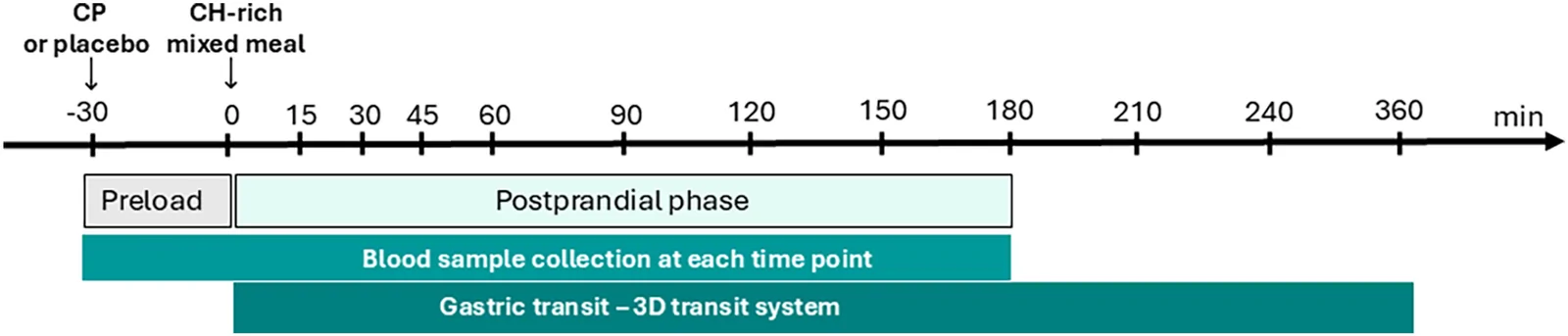
Schematic illustration of the study protocol. Overview of blood sampling timepoints and gastric transit assessment during the preload and postprandial phases of the meal test. CH, carbohydrates; CP, collagen peptide.

The experimental procedure was conducted in 5 distinct steps to ensure precise timing of the preload and postprandial phases (overview Figure 1 for illustration):

1. A baseline blood sample was collected at time point −30 min, marking the start of the preload phase.
2. Immediately after this initial sampling, participants consumed the study product solution (CP or placebo).
3. Thirty minutes later, another sample was collected at time point 0 min.
4. Immediately after the 0-min sample, participants began consuming a standardized, carbohydrate-rich mixed meal with 50 mL of water, which they were instructed to finish within 10 min. This marked the start of the postprandial phase.
5. Upon completion of the mixed meal, the capsule for measuring gastric transit (3-D Transit system, Motilis Medica SA) was ingested with 150 mL of water.

### Blood sampling and laboratory analyses

Venous blood samples were obtained via an indwelling catheter at predefined time points. Samples were collected before meal ingestion (−30 min and 0 min) and repeatedly over a 180-min period thereafter for glucose, insulin, and C-peptide determination, whereas the incretin response with GLP-1 and GIP was measured only over a 120-min period.

Glucose was collected in GlucoEXACT tubes (Sarstedt AG) to ensure complete inhibition of glycolysis in accordance with recommendations for glucose diagnostics. Serum tubes were used for insulin and C-peptide measurements, and EDTA tubes (Sarstedt AG) pretreated with DPP-IV inhibitor (Merck KGaA) were used for incretin analyses. All blood samples were centrifuged at 3000 × *g* for 10 min at 4°C, and aliquots were stored below −70°C until analysis, where applicable.

Glucose, insulin, and C-peptide, as well as hemogram and safety laboratory tests, including liver enzymes, lipid status, and creatinine, were analyzed on the day of sampling by an accredited laboratory (Synlab, MVZ Laboratory). GLP-1 total and GIP total concentrations were measured by ELISA using commercially available kits (EZGLPT-36K and EZHGIP-54K, respectively, Merck KGaA). Incretin analyses were performed by BioTeSys GmbH according to the manufacturer’s instructions.

### Capsule sojourn time and gastric transit

Capsule gastric residence time was assessed using the 3D-Transit system (Motilis Medica SA), a wireless noninvasive electromagnetic capsule system for the evaluation of gastrointestinal motility. The ingested capsule (21.5 × 8.3 mm) is a nondigestible solid; thus, the primary endpoint derived from this method specifically represents the gastric residence time (pyloric passage time) of the capsule itself. During the postprandial fed state, the pylorus preferentially permits liquids and finely ground food particles to transit into the duodenum, whereas larger, nondigestible solids are typically retained in the stomach. Passage of such solids is generally delayed until the late postprandial period or mediated by the return of fasting-state motility, such as phase III migrating motor complex activity [[20], [21], [22], [23]]. Consequently, capsule gastric residence time serves as an index of postprandial gastric motor patterns and the overall duration of the fed state.

The capsule emitted a continuous electromagnetic field detected by multiple sensors housed within a portable, body-worn receiver plate, with raw data stored for offline postprocessing [23,24]. The receiver was worn for a standardized duration of ≥4 h and ≤6 h post ingestion. Recording was terminated after 6 h irrespective of pyloric passage status. Gastric residence time was subsequently determined through region-specific contraction frequencies and spatial coordinate changes, with pyloric passage precisely identified by the characteristic abrupt transition from gastric to duodenal motility patterns and trajectory [21,23]. After complete gastrointestinal transit, the capsule was naturally excreted.

### Outcomes and statistical analysis

The primary endpoint was postprandial glucose incremental area under the curve from 0 to 180 min (iAUC_0-180 min_). Sample size was based on a pilot study [19]. Assuming an effect size of 0.536, corresponding to a mean difference of 750 mg/dL∗min and a SD of 1400 mg/dL∗min between 2 dependent means, 30 participants were required for the crossover design to detect a difference between verum and placebo with 80% power at a 2-sided significance level of 0.05.

iAUC, maximum increase from baseline (Δ*C*_max_), and time to maximum concentration (*T*_max_) were calculated from individual concentration–time curves using the trapezoidal rule. iAUC and Δ*C*_max_ were analyzed using a linear mixed model including treatment, period, sequence, and baseline concentration within the study period as fixed effects and participant as a random effect. Results are presented as least-squares mean differences between CP and placebo with 95% confidence intervals. Statistical analyses were performed using SAS version 9.4. *T*_max_ was evaluated according to [25] with nonparametric test statistics. Percentage changes were calculated from mean values.

Normality of model residuals was assessed using the Shapiro–Wilk test with a significance level of 0.05; log transformation was applied where appropriate, and treatment ratios were presented in such cases.

Prespecified subgroup analyses were planned for participants with prediabetes and normoglycemia. Postprandial glucose response was assessed from 0 to 180 min to account for the inclusion of participants with prediabetes and to allow sufficient time for glucose concentrations to return toward baseline. For insulin and incretin responses, the preload phase from −30 to 0 min was analyzed separately in addition to the overall preload and postprandial period (−30 to 180 min for insulin and C-peptide and −30 to 120 min for the incretin response). For the preload phase within and between interventions, comparisons were performed with paired t tests, or if applicable, the Wilcoxon signed-rank test, and unpaired t tests, or if applicable, the Wilcoxon rank-sum test. To evaluate differences in the evolution of the concentration–time curves between interventions, a 2-way analysis of variance with correction for multiple comparisons according to Šídák was applied.

## Results

### Demographic and baseline characteristics

**FIGURE 2:**
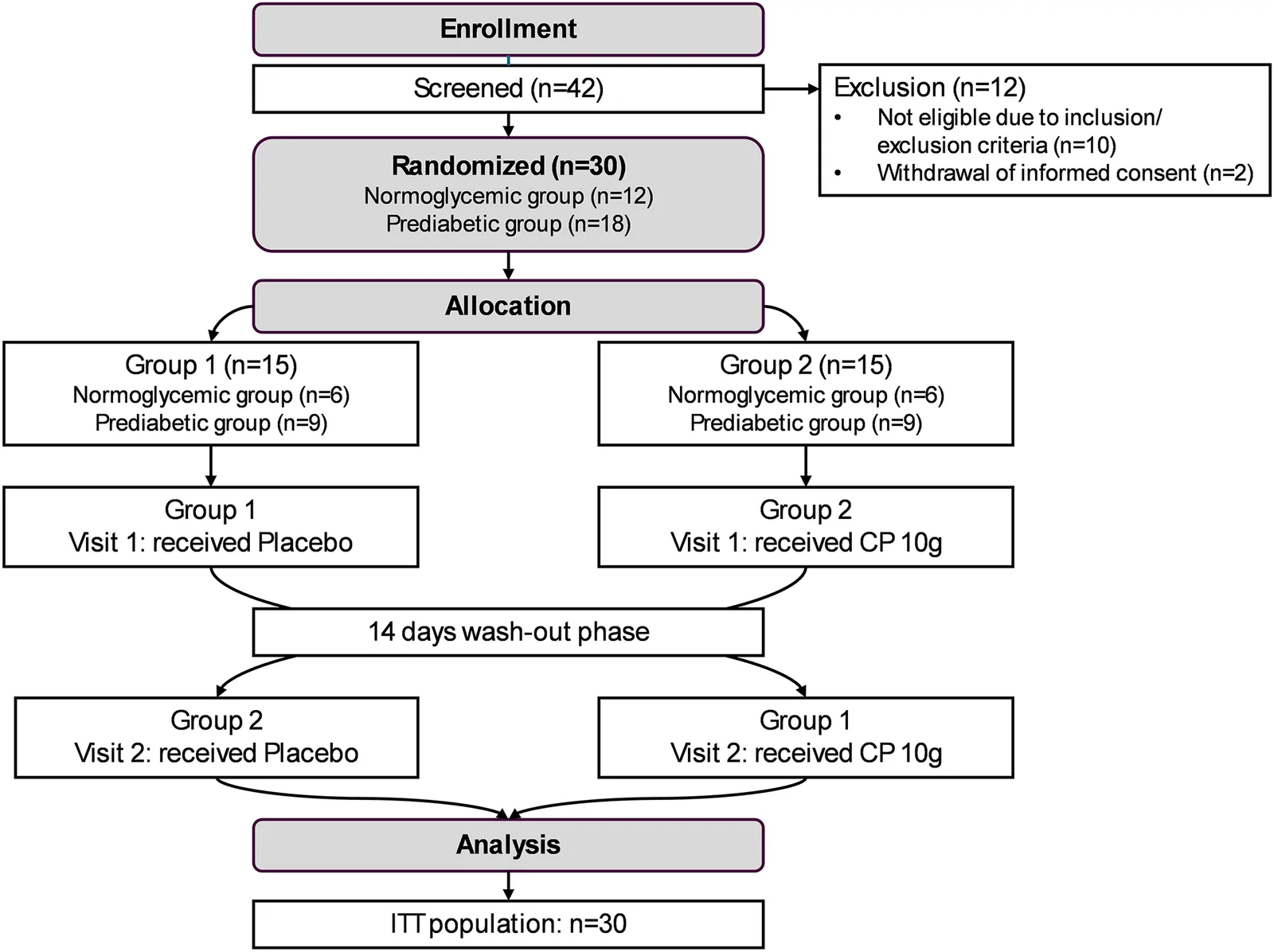
Participant disposition. CP, collagen peptide; ITT, intention to treat.

Forty-two women and men were screened for eligibility, and 30 participants were enrolled. All participants completed the study (participant disposition in Figure 2). The study population comprised 12 men (40%) and 18 women (60%). Four men and 8 women were classified as normoglycemic, whereas 8 men and 10 women were classified as prediabetic.

Baseline demographic characteristics, vital signs, and laboratory values at screening are summarized in Table 1. On average, participants with normoglycemia were slightly younger (*P* = 0.0068), and BMI was significantly lower (*P* = 0.0348) than in the prediabetic subgroup. Furthermore, participants with prediabetes had higher fasting glucose concentrations (*P* = 0.0001) and HbA1c values (*P* = 0.0004) in comparison with normoglycemic. Differences between subgroups were also reflected in baseline insulin concentrations (*P* = 0.0183).

**TABLE 1:** Baseline characteristics in total group and subgroups [mean (95% CI)]

	Total group (*n* = 30)	Normoglycemic subgroup (*n* = 12)	Prediabetic subgroup (*n* = 18)
Age (y)	59.2 (56.7, 61.7)	55.3 (52.5, 58.2)	61.8 (58.5, 65.2)
BMI (kg/m^2^)	25.5 (24.1, 27.0)	23.8 (21.8, 25.7)	26.7 (24.8, 28.7)
SBP (mm Hg)	135.4 (129.1, 141.7)	130.9 (121.0, 140.9)	138.4 (129.8, 146.9)
DBP (mm Hg)	86.8 (83.1, 90.4)	84.8 (78.1, 91.6)	88.1 (83.5, 92.7)
Fasting glucose (mg/dL)	102.8 (99.2, 106.5)	93.1 (89.6, 96.5)	109.3 (106.4, 112.1)
Insulin (μU/mL)	6.0 (4.8, 7.3)	4.3 (2.9, 5.7)	7.2 (5.4, 8.9)
HbA1c (%)	5.46 (5.33, 5.59)	5.21 (5.10, 5.32)	5.63 (5.47, 5.80)
Total cholesterol (mg/dL)	214.3 (201.6, 227.0)	215.8 (196.9, 234.8)	213.3 (194.8, 231.7)
Triglycerides (mg/dL)	102.0 (85.0, 119.1)	86.8 (65.1, 108.6)	112.2 (87.1, 137.2)
GPT (U/L)	22.9 (18.9, 26.8)	22.7 (13.6, 31.7)	23.0 (19.1, 26.9)
GOT (U/L)	25.2 (23.4, 26.9)	26.4 (23.9, 29.0)	24.3 (21.8, 26.9)
Creatinine (mg/dL)	0.85 (0.78, 0.92)	0.83 (0.74, 0.92)	0.86 (0.76, 0.96)

Abbreviations: CI, confidence interval; DBP, diastolic blood pressure; GOT, glutamic oxaloacetic transaminase; GPT, glutamic pyruvic transaminase; HbA1c, hemoglobin A1c; SBP, systolic blood pressure.

### Glycemic response

**FIGURE 3:**
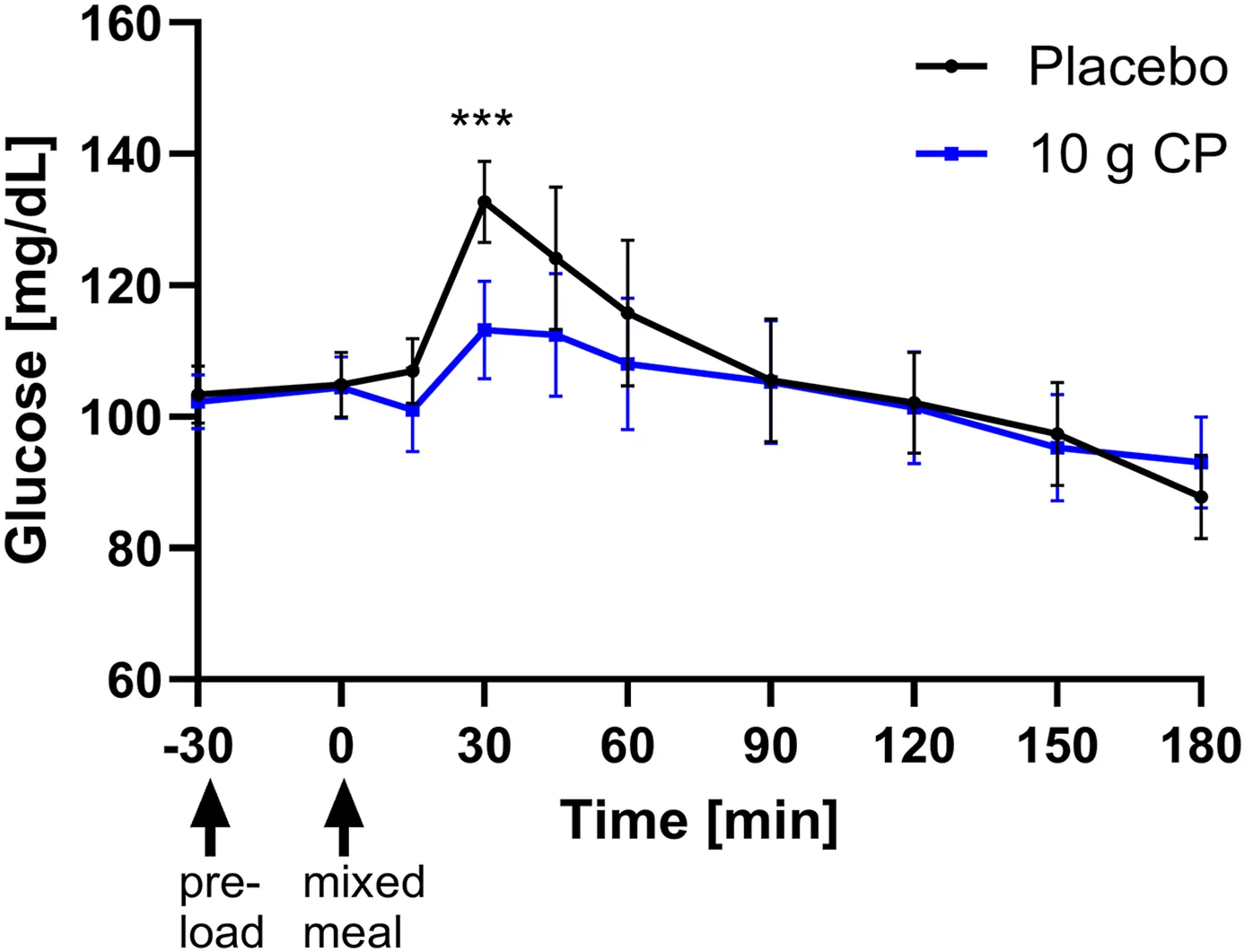
Concentration–time curves of glucose response after intake of study products (−30 min) and mixed meal (0 min) (mean ± 95% CI); ∗∗∗*P* < 0.001. CI, confidence interval; CP, collagen peptide.

Glucose concentration–time profiles showed lower postprandial glucose excursions after 10 g CP ingestion 30 min before the standardized meal (Figure 3).

**FIGURE 4:**
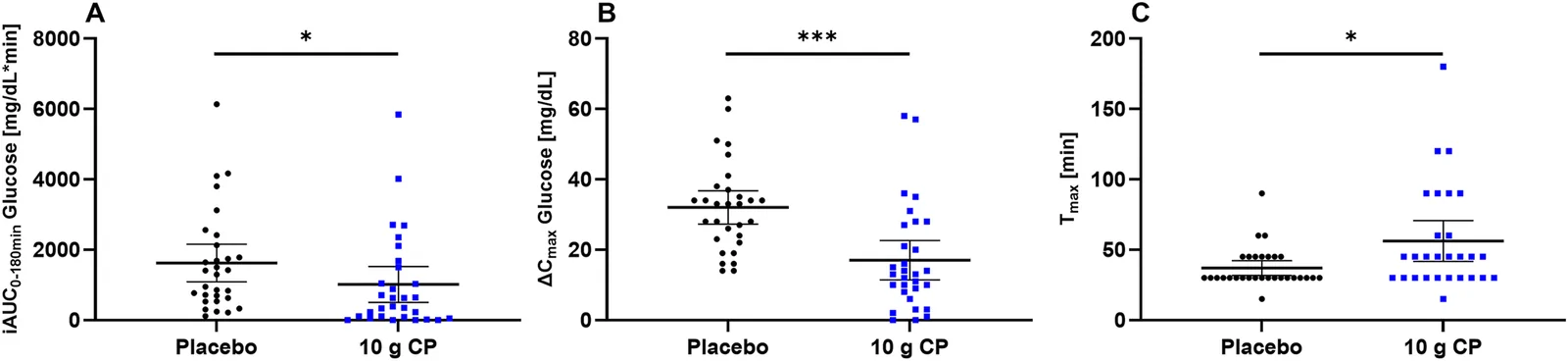
Glycemic response after mixed meal with scatter diagram of pharmacokinetic endpoints with mean ± 95% CI of (A) iAUC_0-180 min_ of glucose, (B) Δ*C*_max_, and (C) *T*_max_; ∗*P* < 0.05; ∗∗∗*P* < 0.0001. Δ*C*_max_, maximum increase in concentration above baseline; CI, confidence interval; CP, collagen peptide; iAUC_0-180 min_, incremental area under the curve from 0 to 180 min.

Compared with placebo, preload ingestion of 10 g CP significantly reduced glucose iAUC, with a least-squares mean difference of −598 mg/dL∗min, *P* = 0.0178, corresponding to a relative reduction of ∼37% (Figure 4A, Table 2).

**TABLE 2:** Glucose iAUC_0-180 min_ and Δ*C*_max_

	Placebo	10 g CP	Treatment difference	*P* value
	LS mean (95% CI)	LS mean (95% CI)	Difference (95% CI)	
Total group (*n* = 30)				
iAUC_0-180 min_ (mg/dL∗min)	1617 (1112, 2123)	1019 (514, 1525)	−598 (−1084, −111)	0.0178
Δ*C*_max_ (mg/dL)	32.0 (27.3, 36.7)	17.1 (12.4, 21.8)	−14.9 (−19.8, −10.0)	<0.0001
Normoglycemic subgroup (*n* = 12)				
iAUC_0-180 min_ (mg/dL∗min)	1192 (582, 1803)	554 (−57, 1165)	−638 (−1172, −104)	0.0237
ΔC_max_ (mg/dL)	27.7 (21.9, 33.5)	11.7 (5.9, 17.5)	−16.0 (−23.0, −9.0)	0.0004
Prediabetic subgroup (*n* = 18)				
iAUC_0-180 min_ (mg/dL∗min)	1911 (1161, 2662)	1319 (568, 2070)	−593 (−1404, 219)	0.1409
Δ*C*_max_ (mg/dL)	34.8 (27.6, 41.9)	20.8 (13.6, 27.9)	−14.0 (−21.4, −6.6)	0.0011

Data are presented as least-squares (LS) means with 95% CI.

Abbreviations: Δ*C*_max_, maximum increase in concentration above baseline; CI, confidence interval; CP, collagen peptide; iAUC_0-180 min_, incremental area under the curve from 0 to 180 min.

As residuals of the linear mixed model were not normally distributed, a sensitivity analysis using the Wilcoxon signed-rank test was performed and confirmed the result (*P* = 0.0099). Peak postprandial glucose excursion was also significantly reduced after CP intake, with a mean intervention difference in Δ*C*_max_ of −14.9 mg/dL, *P* < 0.0001, corresponding to a ∼47% reduction compared with placebo (Figure 4B, Table 2).

*T*_max_ was significantly delayed after CP ingestion with an average delay of 19 min (*P* = 0.0124, Figure 4C).

Subgroup analyses showed that attenuation of postprandial glycemia was present in participants with normoglycemia and those with prediabetes (Table 2). In normoglycemics, iAUC and Δ*C*_max_ were significantly reduced after preload with 10 g CP in comparison with placebo. In the prediabetic subgroup, the reduction in glucose iAUC did not reach statistical significance, whereas the reduction in Δ*C*_max_ was significant. Furthermore, a significant delay in *T*_max_ was confirmed for the prediabetic (*P* = 0.0095) but not the normoglycemic subgroup (*P* = 0.8815) (data not shown).

### Preload phase

#### Insulin and C-peptide

**FIGURE 5:**
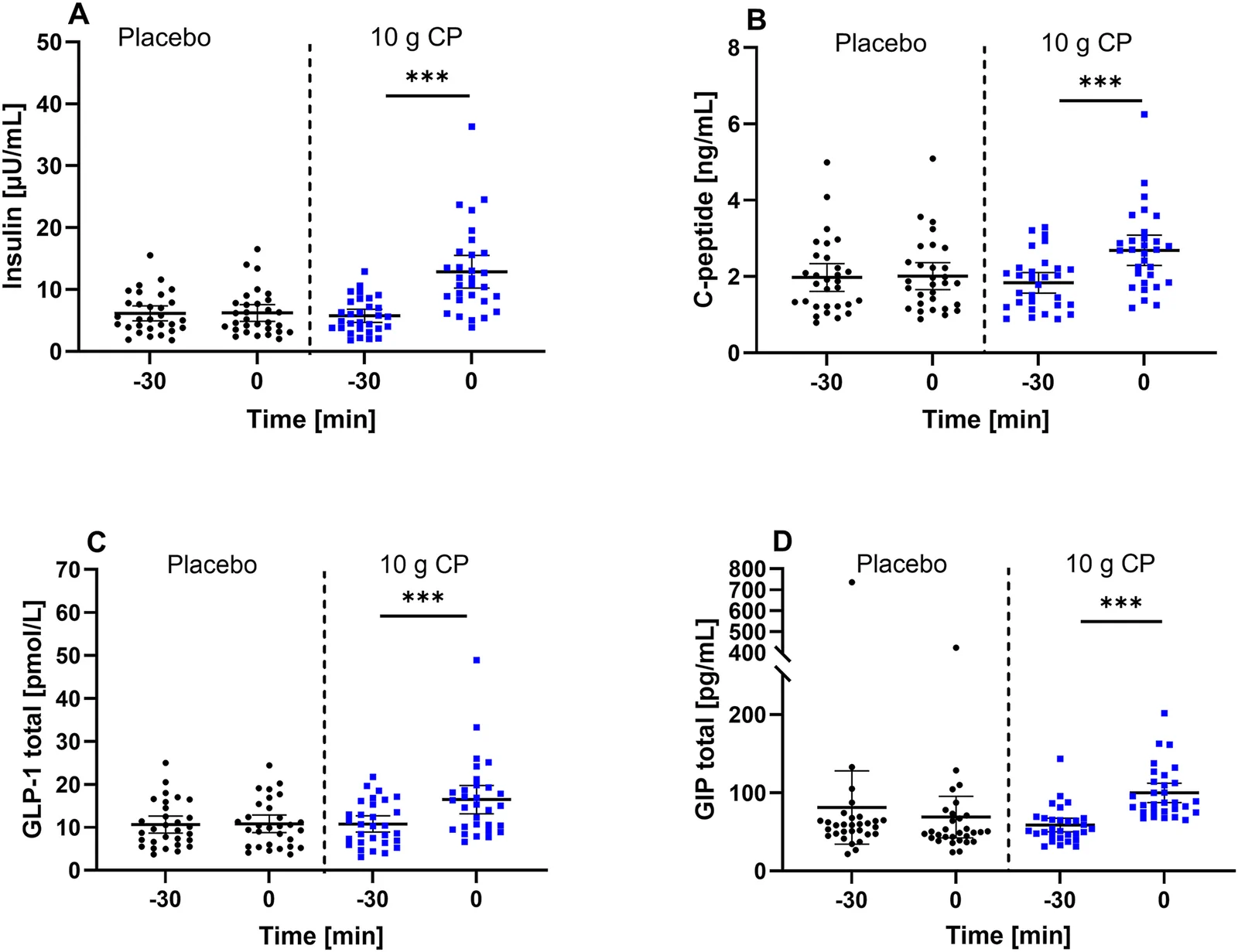
Scatter diagram with mean ± 95% CI of (A) insulin, (B) C-peptide, (C) GLP-1 total, and (D) GIP total in the preload phase with intake of the study products, placebo or 10 g CP at −30 min; ∗∗∗*P* < 0.0001. CI, confidence interval; CP, collagen peptide; GIP, glucose-dependent insulinotropic polypeptide; GLP-1, glucagon-like peptide-1.

During the preload phase, ingestion of 10 g CP significantly increased insulin and C-peptide concentrations compared with placebo (both *P* < 0.0001; Figure 5A and B). Changes from baseline were significantly greater after 10 g CP than placebo (Δinsulin: placebo 0.07 compared with 10 g CP 7.10 μU/mL, *P* < 0.0001; ΔC-peptide: placebo 0.04 compared with 10 g CP 0.85 ng/mL, *P* < 0.0001, Supplemental Figure 1A,B). Similar effects were observed in participants with normoglycemia (Δinsulin: 0.29 compared with 5.66 μU/mL, *P* < 0.0001; ΔC-peptide: 0.07 compared with 0.67 ng/mL, *P* < 0.0001 for delta changes between placebo and 10 g CP, respectively) and participants with prediabetes (Δinsulin: −0.78 compared with 8.07 μU/mL, *P* < 0.0001; ΔC-peptide: 0.01 compared with 0.97 ng/mL, *P* < 0.0001 for delta changes between placebo and 10 g CP, respectively) (Supplemental Figures 2A,B and 3A,B).

#### Incretins GLP-1 total and GIP total

CP induced a marked increase in GLP-1 total concentrations during the 30-min preload phase, whereas GLP-1 total remained near baseline after placebo intake (Figure 5C). Mean GLP-1 total concentrations increased from 10.8 to 16.5 pmol/L (*P* < 0.0001) after CP intake, corresponding to an increase of ∼53%. Changes from baseline were significantly greater with CP than placebo (0.2 compared with 5.7 pmol/L; *P* < 0.0001, Supplemental Figure 1C). Similar responses were observed in participants with normoglycemia (−0.2 compared with 5.2 pmol/L, *P* = 0.0002) and those with prediabetes (0.4 compared with 6.0 pmol/L, *P* < 0.0001; Supplemental Figures 2C and 3C).

A significant rise in GIP total was also observed during the preload phase (Figure 5D**)**. CP increased GIP total concentrations by 40.9 pg/mL, *P* < 0.0001, corresponding to an increase of ∼70%, whereas placebo was associated with a numerical decrease of 12.3 pg/mL (*P* = 0.0571). The difference in change from baseline between interventions was significant (*P* < 0.0001, Supplemental Figure 1D). Similar increases were observed in both subgroups after CP in comparison with placebo (normoglycemic: 36.9 compared with 0.2 pg/mL, *P* = 0.0269; prediabetic: 43.7 compared with −20.7 pg/mL, *P* < 0.0001, Supplemental Figures 2D and 3D).

### Concentration–time curves

#### Insulin and C-peptide response

**FIGURE 6:**
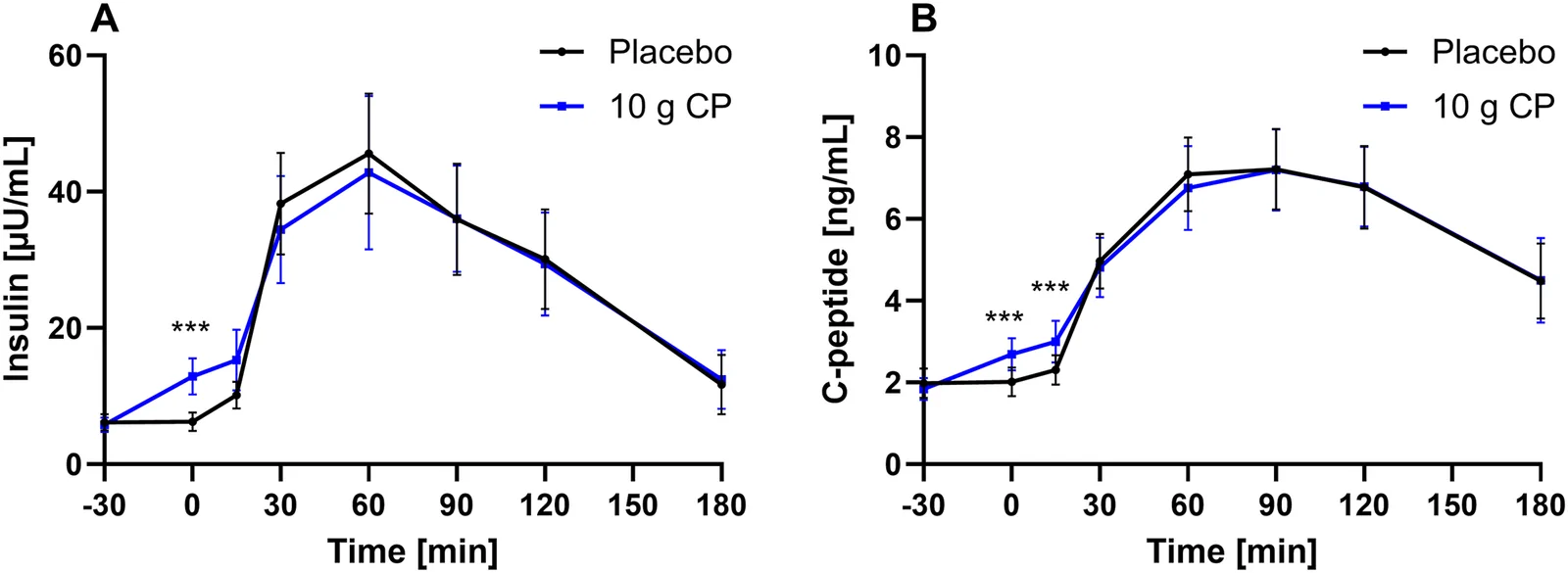
Concentration–time curves of (A) insulin and (B) C-peptide response after intake of study products (−30 min) and mixed meal (0 min) (mean ± 95% CI); ∗∗∗*P* < 0.001. CI, confidence interval; CP, collagen peptide.

**FIGURE 7:**
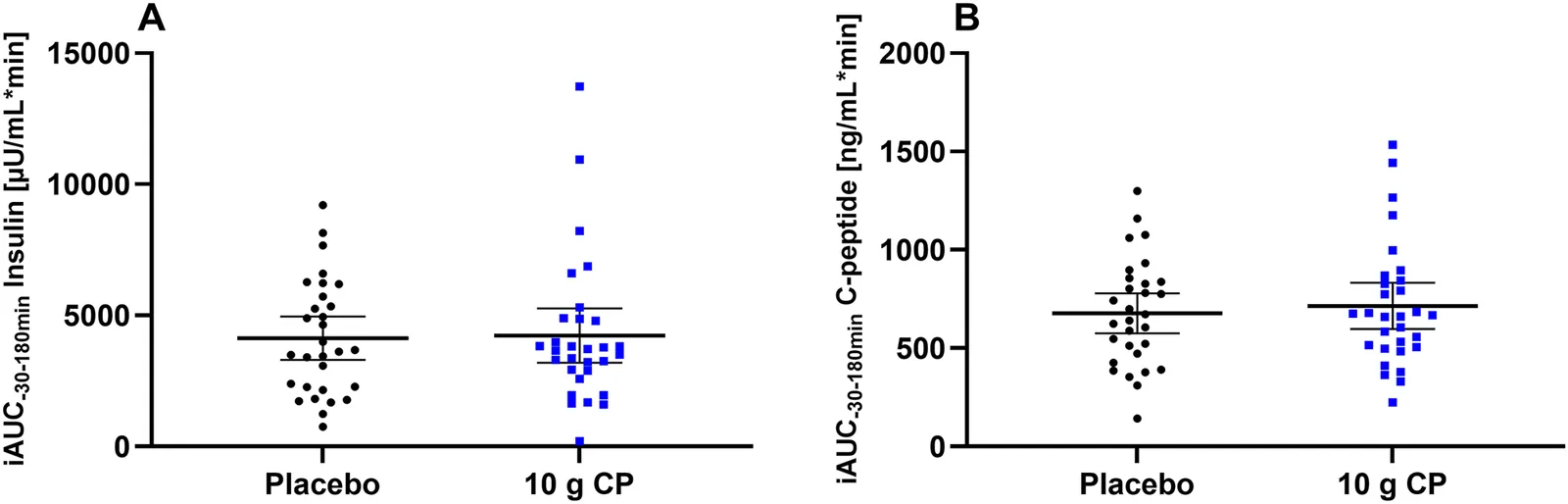
Scatter diagram with mean ± 95% CI of iAUC_-30-180 min_ of (A) insulin (μU/mL∗min) and (B) C-peptide (ng/mL∗min) after intake of study products (−30 min) and mixed meal (0 min). CI, confidence interval; CP, collagen peptide; iAUC_-30-180 min_, incremental area under the curve from −30 to 180 min.

The insulin concentration–time curves were higher after CP administration compared with placebo during the preload phase (Figure 6A), but were comparable between conditions in response to the mixed meal. Across the observation period from −30 to 180 min, total insulin exposure iAUC did not significantly differ between CP and placebo (Figure 7A). C-peptide findings largely paralleled those of insulin, as shown in FIGURE 6, FIGURE 7B. The results were also confirmed in normoglycemic and prediabetic subgroups, as shown in TABLE 3, TABLE 4. Concentration–time curves of subgroups are shown in Supplemental Figures 4A,B, and 5A,B.

**TABLE 3:** Insulin iAUC_-30-180 min_

Insulin iAUC_-30-180 min_ (μU/mL∗min)	Placebo	10 g CP	Treatment ratio	*P* value
	LS mean (95% CI)	LS mean (95% CI)	Ratio (95% CI)	
Total group (*n* = 30)	3465 (2736, 4388)	3500 (2763, 4432)	1.01 (0.87, 1.17)	0.8929
Normoglycemic subgroup (*n* = 12)	3287 (2411, 4481)	3356 (2462, 4576)	1.02 (0.85, 1.23)	0.8076
Prediabetic subgroup (*n* = 18)	3581 (2502, 5125)	3606 (2519, 5161)	1.01 (0.79, 1.29)	0.9530

Data are presented as least-squares (LS) means with 95% CI, and treatment ratios are based on log-transformed data.

Abbreviations: CI, confidence interval; CP, collagen peptide; iAUC_-30-180 min_, incremental area under the curve from −30 to 180 min.

**TABLE 4:** C-peptide iAUC_−30-180 min_

C-Peptide iAUC_-30-180 min_ (ng/mL∗min)	Placebo	10 g CP	Treatment difference	*P* value
	LS mean (95% CI)	LS mean (95% CI)	Difference (95% CI)	
Total group (*n* = 30)	671 (569, 773)	721 (619, 823)	50.0 (−27.8, 128)	0.1984
Normoglycemic subgroup (*n* = 12)	638 (472, 804)	631 (465, 796)	−7.2 (−110, 95.8)	0.8796
Prediabetic subgroup (*n* = 18)	693 (548, 837)	782 (637, 926)	89.1 (−31.4, 210)	0.1357

Data are presented as least-squares (LS) means with 95% CI.

Abbreviations: CI, confidence interval; CP, collagen peptide; iAUC_-30-180 min_, incremental area under the curve from −30 to 180 min.

*T*_max_ of insulin was not significantly altered by CP, although a slight descriptive delay was observed (placebo: 62 min; 10 g CP: 67 min, *P* = 0.3732). Consistent with this finding, CP significantly delayed C-peptide T_max_ after the mixed meal by ∼15 min (placebo: 78 min, 10 g CP: 93 min, *P* = 0.0192), in line with the delayed glucose peak. A similar trend for a delay of 15 min was observed in both subgroups, although statistical significance was not reached in the subgroup analyses (normoglycemic: *P* = 0.0750; prediabetic: *P* = 0.1175).

#### GLP-1 total and GIP total response

**FIGURE 8:**
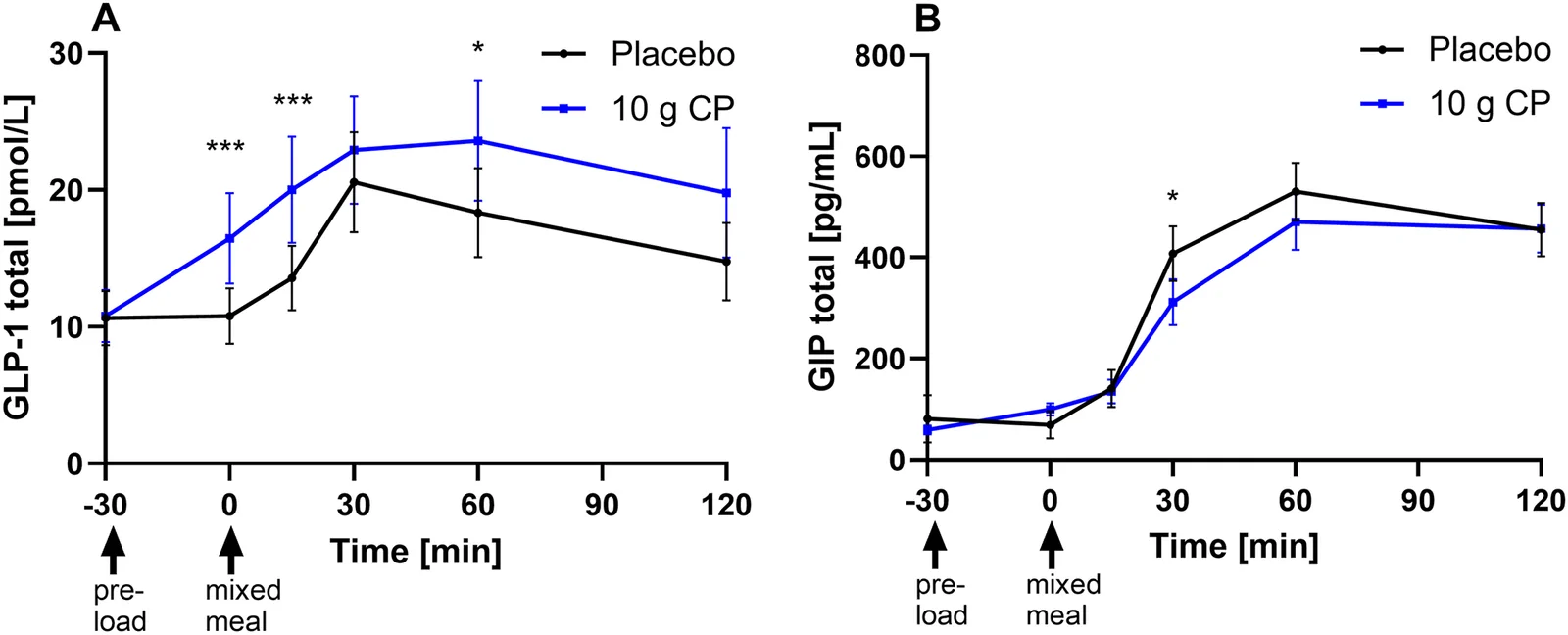
Concentration–time curves of (A) GLP-1 total and (B) GIP total response after intake of study products (–30 min) and mixed meal (0 min) (mean ± 95% CI); ∗*P* < 0.05, ∗∗∗*P* < 0.001. CI, confidence interval; CP, collagen peptide; GIP, glucose-dependent insulinotropic polypeptide; GLP-1, glucagon-like peptide-1.

Concentration–time curves suggested a sustained elevation of GLP-1 total after CP intake, with mean concentrations exceeding placebo at all measured time points (Figure 8A).

**FIGURE 9:**
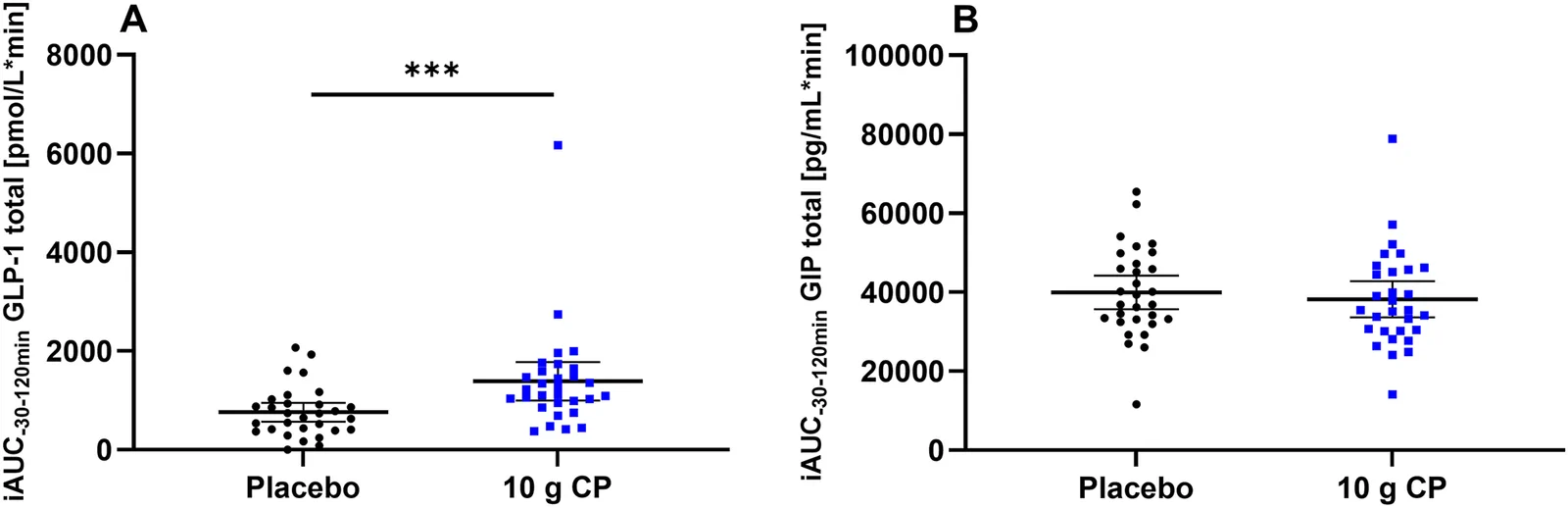
Scatter diagram with mean ± 95% CI of iAUC_-30-180 min_ of (A) GLP-1 total and (B) GIP total after intake of study products (−30 min) and mixed meal (0 min); ∗∗∗*P* < 0.001. CI, confidence interval; CP, collagen peptide; GIP, glucose-dependent insulinotropic polypeptide; GLP-1, glucagon-like peptide-1; iAUC_-30-120 min_, incremental area under the curve from −30 to 120 min; iAUC_-30-180 min_, incremental area under the curve from −30 to 180 min.

GLP-1 total exposure was significantly greater after 10 g CP than placebo, corresponding to a ∼82% higher iAUC (Figure 9A). This effect was also present in both subgroups (Table 5). After the mixed meal, GIP total concentrations increased markedly under both conditions (Figure 8B). However, no significant differences for GIP total exposure were observed between 10 g CP and placebo (Figure 9B, Table 6). Concentration–time curves of subgroups are shown in Supplemental Figures 4C,D, and 5C,D.

**TABLE 5:** GLP-1 total iAUC_-30-120 min_

GLP-1 total iAUC_-30-120 min_ (pmoL/L∗min)	Placebo	10 g CP	Treatment ratio	*P* value
	LS mean (95% CI)	LS mean (95% CI)	Ratio (95% CI)	
Total group (*n* = 30)	633 (498, 805)	1166 (921, 1476)	1.84 (1.37, 2.48)	0.0002
Normoglycemic subgroup (*n* = 12)	688 (471, 1005)	1347 (922, 1968)	1.96 (1.36, 2.82)	0.0020
Prediabetic subgroup (*n* = 18)	608 (430, 861)	1053 (753, 1474)	1.73 (1.06, 2.82)	0.0293

Data are presented as least-squares (LS) means with 95% CI, and treatment ratios are based on log-transformed data.

Abbreviations: CI, confidence interval; CP, collagen peptide; GLP-1, glucagon-like peptide-1; iAUC_-30-120 min_, incremental area under the curve from −30 to 120 min.

**TABLE 6:** GIP total iAUC_-30-120 min_

GIP total iAUC_-30-120 min_ (pg/mL∗min)	Placebo	10 g CP	Treatment difference	*P* value
	LS mean (95% CI)	LS mean (95% CI)	Difference (95% CI)	
Total group (*n* = 30)	40,128 (35,760, 44,497)	38,012 (33,644, 42,381)	−2116 (−5977, 1745)	0.2707
Normoglycemic subgroup (*n* = 12)	44,775 (36,680, 52,869)	40,798 (32,703, 48,892)	−3977 (−12,432, 4478)	0.3177
Prediabetic subgroup (*n* = 18)	36,915 (31,732, 42,099)	36,270 (31,087, 41,454)	−645 (−4927, 3637)	0.7528

Data are presented as least-squares (LS) means with 95% CI.

Abbreviations: CI, confidence interval; CP, collagen peptide; GIP, glucose-dependent insulinotropic polypeptide; iAUC_-30-120 min_, incremental area under the curve from −30 to 120 min.

Both GLP-1 total and GIP total showed delayed *T*_max_ following 10 g CP compared with placebo. For GLP-1 total, the delay did not reach statistical significance (*T*_max_: placebo 43.5 min; 10 g CP: 54.5, *P* = 0.1033), whereas the *T*_max_ of GIP total was significantly delayed by ∼17 min (*T*_max_: placebo: 69.0 min, 10 g CP: 86.0 min; *P* = 0.0259). This delay in GIP total was evident in both subgroups but reached statistical significance only in the prediabetic subgroup (normoglycemic: placebo: 67.5 min; 10 g CP: 80 min; *P* = 0.3002; prediabetic: placebo: 70 min; 10 g CP: 90 min; *P* = 0.0455).

### Capsule gastric residence time

**FIGURE 10:**
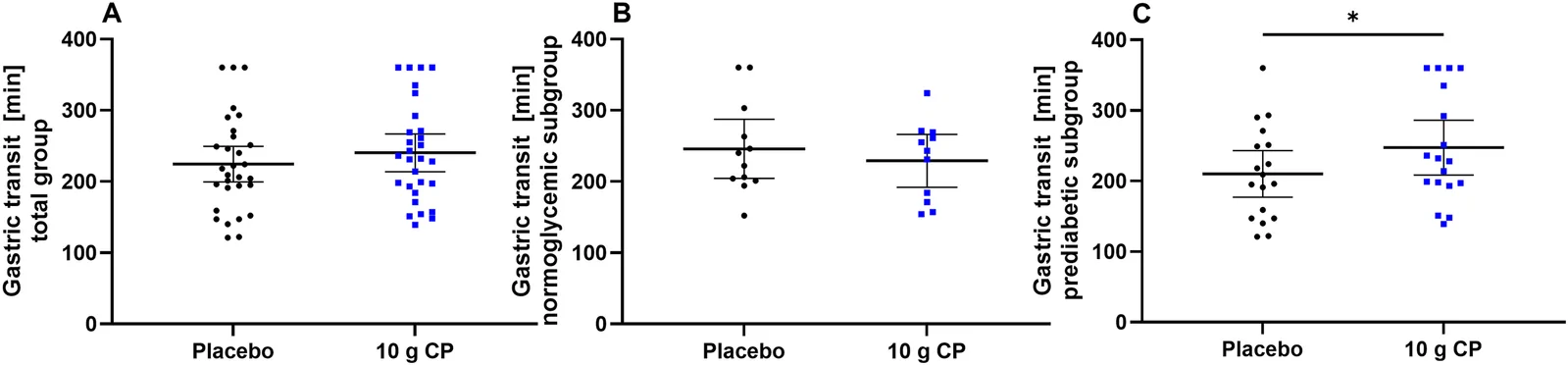
Distribution of gastric transit of the capsule (min) after intake of the mixed meal (0 min) in (A) total group, (B) normoglycemic subgroup, and (C) prediabetic subgroup (Scatter diagram with mean ± 95% CI); ∗*P* < 0.05. CI, confidence interval; CP, collagen peptide.

Overall, in 52 of 60 assessments (87%), the capsule passed through the pylorus within 6 h. Mean gastric residence time of the capsule in the study population was 3.7 h. In the total cohort, capsule gastric residence time showed a nonsignificant trend toward being prolonged after the ingestion of 10 g CP preload compared with placebo (mean difference: 20.7 min; Figure 10, Table 7). Subgroup analyses showed no relevant effect in participants with normoglycemia, whereas pyloric passage of the capsule was significantly delayed in participants with prediabetes by 37.2 min (*P* = 0.0436; Figure 10).

**TABLE 7:** Gastric transit as assessed by residence time of the capsule

Gastric residence time of the capsule (min)	Placebo	10 g CP	Treatment difference	*P* value
	LS mean (95% CI)	LS mean (95% CI)	Difference (95% CI)	
Total group (*n* = 30)	224.5 (198.8, 250.1)	242.5 (216.5, 268.5)	18.0 (−8.7, 44.7)	0.1774
Normoglycemic subgroup (*n* = 12)	245.9 (205.5, 286.3)	235.5 (193.8, 277.2)	−10.4 (−51.6, 30.8)	0.5793
Prediabetic subgroup (*n* = 18)	210.2 (174.8, 245.5)	247.4 (212.0, 282.8)	37.2 (1.2, 73.2)	0.0436

Data are presented as least-squares (LS) means with 95% CI.

Abbreviations: CI, confidence interval; CP, collagen peptide.

### Safety and tolerability

The study products were well tolerated, with no serious adverse events (AEs) reported. A total of 16 mild, unrelated AEs (most frequently headaches and common colds) occurred in 10 participants. These events did not impact study adherence, and all participants completed the protocol successfully.

## Discussion

This study demonstrates that a 10 g preload of specific CP beneficially modulates postprandial glycemic and hormonal responses in both participants with normoglycemia and individuals with impaired glucose regulation. The primary finding is a significant attenuation of the glucose excursion without a compensatory increase in total insulin secretion, suggesting a more efficient postprandial glucose regulation. Supported by prior in vitro data [19], it is hypothesized that CP act locally in the gut lumen to stimulate GLP-1 without inducing an excessive insulinogenic response. The 30-min preload interval aligns with the gastric emptying kinetics of a 200 mL liquid [26], a time window that is proposed to allow bioactive peptides to reach the proximal small intestine and bind to L-cell nutrient sensors, thereby triggering endogenous GLP-1 release before meal ingestion.

Consistent with this mechanism, CP preload stimulated a premeal, feed-forward activation of the incretin hormones GLP-1 and GIP, which was accompanied by a modest, anticipatory release of insulin. This contrasts with the more reactive overall insulin response in the placebo condition after the mixed meal and suggests that CP might promote a more coordinated metabolic adaptation, preparing the organism for the impending nutrient exposure and facilitating more efficient postprandial glucose handling. Interestingly, our data show that the observed improvement in glycemic control was achieved without a compensatory increase in total postprandial insulin secretion (iAUC_-30-180 min_ insulin and C-peptide). In this study, C-peptide responses closely paralleled those observed for insulin. Although C-peptide does not exert a direct metabolic effect in humans, it serves as a robust clinical marker of pancreatic β-cell function. Consequently, measuring C-peptide allowed a more reliable assessment of endogenous insulin secretion in response to CP intake [27]. In this regard, compared with the placebo, a CP preload elicits an early, incretin-mediated insulin response supported by concurrent C-peptide synthesis. This response, further evidenced by a ∼15-min delay in C-peptide *T*_max_ after the mixed meal, establishes metabolic readiness before meal ingestion. Consequently, it may reduce postprandial glucose levels without eliciting compensatory, disproportionate insulin excursions. Unlike established whey-based preload strategies that primarily leverage a strong insulinotropic effect to lower postprandial glucose [16], the CP preload in this study reduced glycemia without increasing total insulin exposure, suggesting a distinct, insulin-efficient physiological response.

Beyond glycemic control, limiting excessive postprandial insulin secretion is increasingly recognized as critical for cardiometabolic health, as chronic hyperinsulinemia is strongly linked to insulin resistance and cardiovascular disease risk [28,29]. The metabolic priming effect suggested by our findings may contribute to the observed blunted and delayed postprandial glucose peak [30,31]. This supports the hypothesis that CP might act as a nutrient-sensing modulator, initiating a proactive incretin and insulin response that optimizes glycemic handling [32,33].

A detailed analysis of the incretin responses showed that CP ingestion led to a sustained and significantly higher elevation of total GLP-1 throughout the postprandial period. In contrast, although GIP was stimulated during the preload phase, its overall postprandial response did not differ from the placebo [34]. The sustained GLP-1 response is consistent with its dual role in both stimulating insulin secretion and delaying gastric emptying [31,35].

This highlights a crucial bidirectional relationship between gut hormone secretion and gastrointestinal motility because postprandial GIP secretion is closely and directly related to the meal composition, the rate of gastric emptying, and subsequent nutrient delivery to the small intestine [36,37]. Consequently, the observed delayed T_max_ for GIP further supports the hypothesis that its secretion profile may have been dictated by the slowed transit and absorption of nutrients. Ultimately, although the early CP-induced GLP-1 may slow gastric transit, this physiological delay could act as a feedback loop that temporally modulates the subsequent release of GIP.

The potential for synergistic GLP-1/GIP signaling is an area of emerging evidence [38,39]. Because both incretins can exert a major impact on glucagon secretion, future research elucidating the interplay of these incretins with CP and their subsequent effect on glucagon dynamics remains an important focus [40]. The distinct incretin dynamics observed here suggest that CP, at a dose of 10 g, might primarily leverage the GLP-1 pathway for its postprandial benefits, thereby not excluding a role for GIP during the preload phase. This aligns with clinical studies demonstrating that premeal nutritional interventions can successfully augment endogenous GLP-1, slow gastric emptying, and support healthy postprandial glycemic control [41,42]. By showing that a standalone 10 g CP preload independently supports sustained GLP-1 elevation and delayed gastric transit in prediabetics, our study supports these nutritional strategies and argues for the physiological potential of targeting early luminal nutrient-sensing pathways. Physiologically, the observed delay in gastric transit represents a critical regulatory mechanism, showing a particularly pronounced effect in the prediabetic subgroup. This finding is of high physiological significance, as recent literature demonstrates that gastric emptying is often abnormally accelerated in individuals with prediabetes and uncomplicated type 2 diabetes, a phenomenon that actively exacerbates postprandial glycemic excursions [43]. By potentially slowing down the transit of the meal from the stomach to the small intestine, CP preload may reduce the rate of glucose appearance in the circulation, which may directly contribute to the observed lower and delayed glucose peak. This finding aligns with the known physiological effects of GLP-1 [26], which was significantly elevated after CP ingestion. It also suggests that individuals with impaired glucose regulation may be particularly responsive to the transit-slowing properties of CP, as further supported by the significant delay in time to reach glucose C_max_ in the prediabetic subgroup. However, because the glucose-lowering effect was observed in both metabolic subgroups, whereas delayed gastric transit reached significance only in participants with prediabetes, additional physiological pathways beyond measurable transit delays are likely involved.

In evaluating these findings, it must be noted that the 3D-Transit capsule passage serves as an index of postprandial fed-state duration and gastric motor behavior rather than a direct measurement of liquid meal emptying rates [23,24]. Although the prolonged capsule residence time after CP, particularly in the prediabetic subgroup, is consistent with an extended fed-state motor pattern, a direct causal delay in nutrient clearance cannot be definitively inferred. Future studies combining CP with validated meal-based emptying techniques are warranted to correlate physical transit with systemic metabolic responses [44].

Altogether, this study demonstrates that a 10 g preload of CP beneficially modulates postprandial glycemic and hormonal responses in participants with normoglycemia and those with prediabetes. This metabolic balance seems to be driven by a coordinated, multifactorial mechanism involving premeal incretin stimulation, a subsequent prolongation of gastric transit, particularly in individuals with prediabetes, and a more coordinated metabolic adaptation to the nutrient load. As reducing postprandial hyperglycemia through dietary strategies is a critical component of metabolic health [36,45], these findings point to CP as an effective strategy to support metabolic balance.

Although this study’s strengths include its power-calculated, randomized, double-blind, placebo-controlled, crossover design, several limitations should be acknowledged. First, this trial evaluated the acute effects of a single CP preload; further research is needed to determine whether these benefits are sustained with chronic daily intake. Second, although the overall sample size was sufficiently powered for the primary endpoint, future studies with larger cohorts are required to enable fully powered, sex-stratified analyses, especially given the established influence of biological sex on these physiological pathways [46]. Third, active incretin concentrations were not measured. Finally, given the close physiological interaction between glucagon and the incretin system [43], the absence of glucagon measurements represents an additional limitation. Future clinical studies incorporating glucagon dynamics and involving direct, liquid-phase nutrient emptying techniques will be valuable to comprehensively map the physiological potential of CP across diverse populations.

In conclusion, the findings of this study suggest that a preload with specific CP may play a role in supporting postprandial glucose control. By stimulating an early incretin response and being associated with prolonged gastric residence time of an indigestible capsule, particularly in prediabetic individuals, this intervention attenuates the postmeal glucose fluctuation. Notably, this acutely improved glycemic control is achieved without increasing the overall insulin burden, highlighting the potential of CP as a valuable nutritional strategy for managing postprandial glycemic regulation and supporting metabolic balance.

## Author contributions

The authors’ responsibilities were as follows – NV, JP, CS: designed research and primarily responsible for final content; NS, CS: conducted research; CS, NS, MW, VS: analyzed data; NV, CIFS, EG, JP: wrote the paper; and all authors: read and approved the final manuscript.

## Data availability

The data supporting this study are presented in the figures and tables. Because of ethical, confidentiality, and patenting constraints, the raw data are not publicly available. Data may be provided by the corresponding author on reasonable request.

## Patents

The research is associated with a patent on the use of hydrolyzed collagen for reducing blood glucose (International Publication No. WO2023233008A1). Filed by Rousselot 391 BV, the patent claims the production process and use of a specific hydrolyzed collagen 392 (Nextida GC) for this purpose.

## Declaration of generative AI and AI-assisted technologies in the writing process

The authors declare that no generative AI or AI-assisted technologies were used in the writing of this manuscript.

## Funding

This research received no external funding. The study was funded by Rousselot BV, Belgium. The sponsor had no influence on the conduct and results of the study. The study was independently performed by BioTeSys GmbH.

## Conflict of interest

CS, NS, VS, and MW declare no conflict of interest. NV, EG, CIFS, and JP are employees of Rousselot BV.

## References

[1] Gerich J.E. (2006). Postprandial hyperglycemia and cardiovascular disease. Endocr. Pract..

[2] Fazio S., Mercurio V., Tibullo L., Fazio V., Affuso F. (2024). Insulin resistance/hyperinsulinemia: an important cardiovascular risk factor that has long been underestimated. Front. Cardiovasc. Med..

[3] Muijs L.T., Racca C., de Wit M., Brouwer A., Wieringa T.H., de Vries R. (2021). Glucose variability and mood in adults with diabetes: a systematic review, Endocrinol. Diabetes Metab..

[4] Penckofer S., Quinn L., Byrn M., Ferrans C., Miller M., Strange P. (2012). Does glycemic variability impact mood and quality of life?. Diabetes Technol. Ther.

[5] Wang Y., Fang Y., Yang G., Prattichizzo F., Ceriello A. (2025). Postprandial 2-h glucose tolerance is associated with diabetes diagnosis, diabetes mortality, and cardiovascular mortality. Sci. Rep..

[6] Mann B.K., Bhandohal J.S., Hong J. (2019). An overall glance of evidence supportive of one-hour and two-hour postload plasma glucose levels as predictors of long-term cardiovascular events. Int. J. Endocrinol..

[7] Seino Y., Fukushima M., Yabe D. (2010). GIP and GLP-1, the two incretin hormones: similarities and differences. J. Diabetes Investig..

[8] Hjørne A.P., Modvig I.M., Holst J.J. (2022). The sensory mechanisms of nutrient-induced GLP-1 secretion. Metabolites.

[9] Alsalim W., Lindgren O., Ahrén B. (2023). Glucose-dependent insulinotropic polypeptide and glucagon-like peptide-1 secretion in humans: characteristics and regulation. J. Diabetes Investig..

[10] Müller T.D., Adriaenssens A., Ahrén B., Blüher M., Birkenfeld A.L., Campbell J.E. (2025). Glucose-dependent insulinotropic polypeptide (GIP). Mol. Metab..

[11] Nauck M.A., Meier J.J. (2016). The incretin effect in healthy individuals and those with type 2 diabetes: physiology, pathophysiology, and response to therapeutic interventions. Lancet Diabetes Endocrinol.

[12] Pederson R.A., Satkunarajah M., McIntosh C.H., Scrocchi L.A., Flamez D., Schuit F. (1998). Enhanced glucose-dependent insulinotropic polypeptide secretion and insulinotropic action in glucagon-like peptide 1 receptor -/- mice. Diabetes.

[13] Wu T., Rayner C.K., Young R.L., Horowitz M. (2013). Gut motility and enteroendocrine secretion. Curr. Opin. Pharmacol..

[14] Jalleh R.J., Jones K.L., Rayner C.K., Marathe C.S., Wu T., Horowitz M. (2022). Normal and disordered gastric emptying in diabetes: recent insights into (patho)physiology, management and impact on glycaemic control. Diabetologia.

[15] Holst J.J., Gribble F., Horowitz M., Rayner C.K. (2016). Roles of the gut in glucose homeostasis. Diabetes Care.

[16] Wu T., Rayner C.K., Jones K.L., Horowitz M. (2023). Seize the whey! Whey Preloads for control of postprandial glycemia in metabolic disease. Am. J. Clin. Nutr..

[17] He L., Gao Y., Ju C., Wang X., Zhang Y., Yu Q. (2025). Collagen peptides alleviate hyperglycemia in mice by modulating insulin resistance, glucose metabolism and gut microbiota. Int. J. Biol. Macromol..

[18] Devasia S., Kumar S., Stephena P.S., Inoue N., Sugihara F., Suzuki K. (2018). Double blind, randomized clinical study to evaluate efficacy of 452 collagen peptide as add on nutritional supplement in type 2 diabetes. J.Clin. Nutr. Food Sci.

[19] Grasset E., Briand F., Virgilio N., Schön C., Wilhelm M., Cudennec B. (2024). A specific collagen hydrolysate improves postprandial glucose tolerance in normoglycemic and prediabetic mice and in a first proof of concept study in healthy, normoglycemic and prediabetic humans. Food Sci. Nutr..

[20] Klinge M.W., Sutter N., Mark E.B., Haase A.-.M., Borghammer P., Schlageter V. (2021). Gastric emptying time and volume of the small intestine as objective markers in patients with symptoms of diabetic enteropathy. J. Neurogastroenterol. Motil..

[21] Liao D., Mark E.B., Nedergaard R.B., Wegeberg A.-.M., Brock C., Krogh K. (2022). Contractility patterns and gastrointestinal movements monitored by a combined magnetic tracking and motility testing unit. Neurogastroenterol. Motil..

[22] Sutter N., Klinge M.W., Mark E.B., Nandhra G., Haase A.-.M., Poulsen J. (2020). Normative values for gastric motility assessed with the 3D-transit electromagnetic tracking system. Neurogastroenterol. Motil..

[23] Haase A.M., Gregersen T., Schlageter V., Scott M.S., Demierre M., Kucera P. (2014). Pilot study trialling a new ambulatory method for the clinical assessment of regional gastrointestinal transit using multiple electromagnetic capsules. Neurogastroenterol. Motil..

[24] Worsøe J., Fynne L., Gregersen T., Schlageter V., Christensen L.A., Dahlerup J.F. (2011). Gastric transit and small intestinal transit time and motility assessed by a magnet tracking system. BMC Gastroenterol.

[25] Schumacher M., Schulgen G. (2008).

[26] Mudie D.M., Murray K., Hoad C.L., Pritchard S.E., Garnett M.C., Amidon G.L. (2014). Quantification of gastrointestinal liquid volumes and distribution following a 240 mL dose of water in the fasted state. Mol. Pharm..

[27] Saisho Y. (2016). Postprandial C-peptide to glucose ratio as a marker of β cell function: implication for the management of type 2 diabetes. Int. J. Mol. Sci..

[28] DeFronzo R.A., Abdul-Ghani M.A. (2011). Preservation of β-cell function: the key to diabetes prevention. J. Clin. Endocrinol. Metab..

[29] Chiasson J.-.L., Rabasa-Lhoret R. (2004). Prevention of type 2 diabetes: insulin resistance and beta-cell function. Diabetes.

[30] Guglielmi C., Del Toro R., Lauria A., Maurizi A.R., Fallucca S., Cappelli A. (2017). Effect of GLP-1 and GIP on C-peptide secretion after glucagon or mixed meal tests: significance in assessing B-cell function in diabetes. Diabetes. Metab. Res. Rev..

[31] Edholm T., Degerblad M., Grybäck P., Hilsted L., Holst J.J., Jacobsson H. (2010). Differential incretin effects of GIP and GLP-1 on gastric emptying, appetite, and insulin-glucose homeostasis. Neurogastroenterol. Motil..

[32] Diakogiannaki E., Gribble F.M., Reimann F. (2012). Nutrient detection by incretin hormone secreting cells. Physiol. Behav..

[33] Duca F.A., Waise T.M.Z., Peppler W.T., Lam T.K.T. (2021). The metabolic impact of small intestinal nutrient sensing. Nat. Commun..

[34] Nauck M.A., Quast D.R., Wefers J., Pfeiffer A.F.H. (2021). The evolving story of incretins (GIP and GLP-1) in metabolic and cardiovascular disease: a pathophysiological update. Diabetes Obes. Metab..

[35] Jalleh R.J., Rayner C.K., Hausken T., Jones K.L., Camilleri M., Horowitz M. (2024). Gastrointestinal effects of GLP-1 receptor agonists: mechanisms, management, and future directions. Lancet Gastroenterol. Hepatol..

[36] Wang Z., Huang W., Rasmussen R.S., Horowitz M., Jones K.L., Rayner C.K. (2026). Chronic glycaemic control modulates the relationship between GIP and glucagon secretion following oral and enteral nutrients in type 2 diabetes. Diabetes Obes. Metab..

[37] Christensen M., Vedtofte L., Holst J.J., Vilsbøll T., Knop F.K. (2011). Glucose-dependent insulinotropic polypeptide: a bifunctional glucose-dependent regulator of glucagon and insulin secretion in humans. Diabetes.

[38] Liu Q.K. (2024). Mechanisms of action and therapeutic applications of GLP-1 and dual GIP/GLP-1 receptor agonists. Front. Endocrinol..

[39] Yamanouchi D. (2025). The roles of incretin hormones GIP and GLP-1 in metabolic and cardiovascular health: a comprehensive review. Int. J. Mol. Sci..

[40] Wu T., Rayner C.K., Marathe C.S., Jones K.L., Horowitz M. (2018). Glucagon receptor signalling - backwards and forwards. Expert Opin. Investig. Drugs..

[41] Wu T., Bound M.J., Zhao B.R., Standfield S.D., Bellon M., Jones K.L. (2013). Effects of a D-xylose preload with or without sitagliptin on gastric emptying, glucagon-like peptide-1, and postprandial glycemia in type 2 diabetes. Diabetes Care.

[42] Wu T., Little T.J., Bound M.J., Borg M., Zhang X., Deacon C.F. (2016). A protein preload enhances the glucose-lowering efficacy of vildagliptin in type 2 diabetes. Diabetes Care.

[43] Watson L.E., Xie C., Wang X., Li Z., Phillips L.K., Sun Z. (2019). Gastric emptying in patients with well-controlled type 2 diabetes compared with young and older control subjects without diabetes. J. Clin. Endocrinol. Metab..

[44] Jalleh R.J., Wu T., Umapathysivam M.M., Young R.L., Jones K.L., Marathe C.S. (2026). Impact of gastric emptying and small intestinal carbohydrate absorption on glycemia in health and diabetes: therapeutic implications. Physiol. Rev..

[45] Ruijgrok C., Blaak E.E., Egli L., Dussort P., Vinoy S., Rauh S.P. (2021). Reducing postprandial glucose in dietary intervention studies and the magnitude of the effect on diabetes-related risk factors: a systematic review and meta-analysis. Eur. J. Nutr..

[46] Xiang C., Sun Y., Luo Y., Xie C., Huang W., Jones K.L. (2024). Gastric emptying is slower in women than men with type 2 diabetes and impacts on postprandial glycaemia. Diabetes Obes. Metab..

