# Supplementary material for: Specific Collagen Peptides Support Postprandial Metabolic Health by Modulating Incretin Hormones, Gastric Transit, and Glycemic Control in Participants with Normoglycemia and Prediabetes
Source: Curr Dev Nutr. 2026 Aug 8;10(9):109512. doi: 10.1016/j.cdnut.2026.109512 (PMC13543905; doi:10.1016/j.cdnut.2026.109512)
Supplement: Supplementary file 1 [file mmc1.pdf]

Specific Collagen Peptides Support Postprandial Metabolic Health by Modulating Incretin Hormones, Gastric Transit, and Glycemic Control in Normoglycemic and Prediabetic Participants

Virgilio et al.

Supplementary Material

Supplementary Figure 1

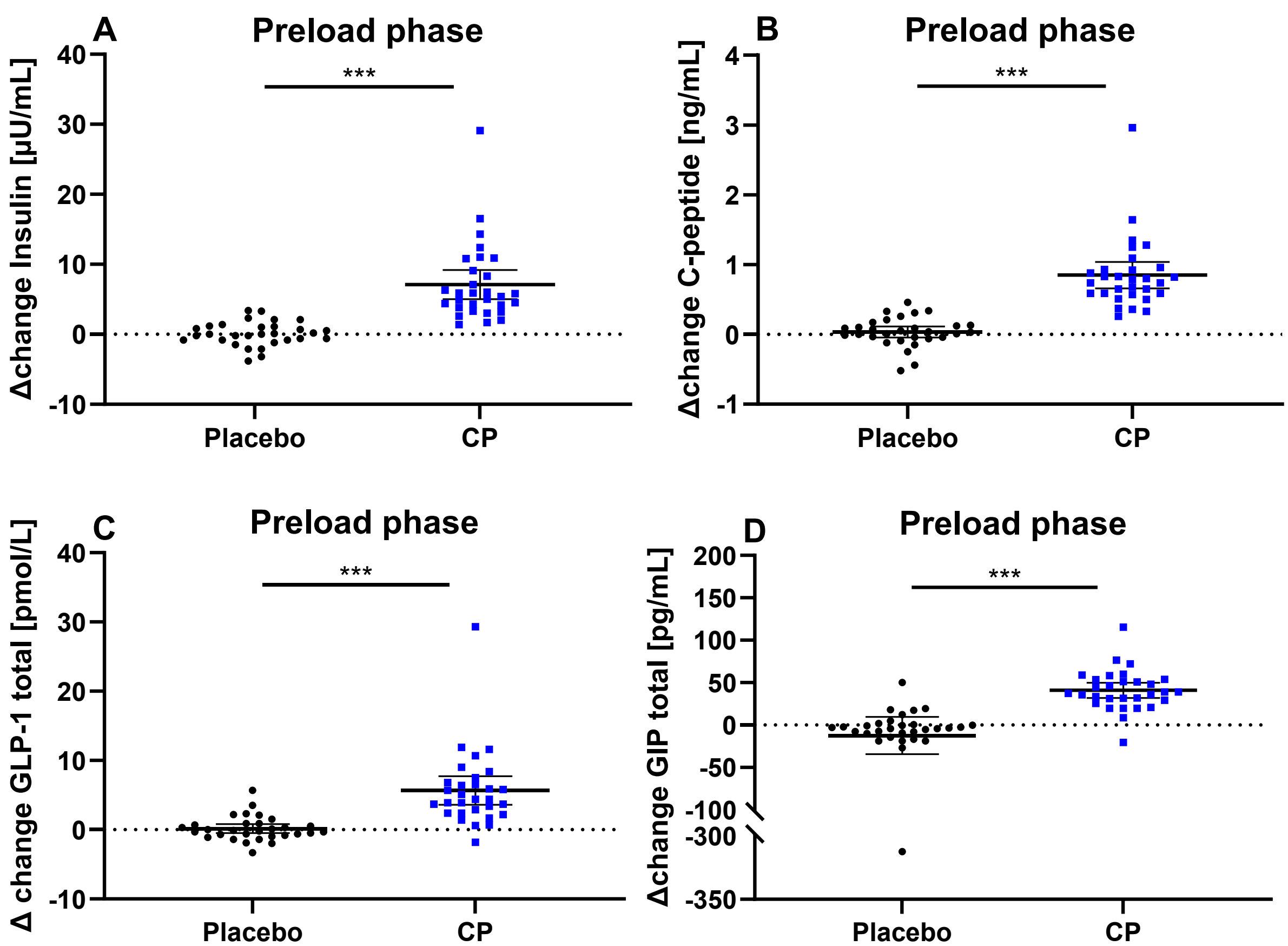

Supplementary Figure S1: Scatter diagram with mean±95% CI for delta changes in preload phase after intake of study products (placebo or 10 g CP) (n=30) of A) insulin, B) C-peptide, C) GLP-1 total and D) GIP total; ***p<0.0001

Supplementary Figure 2

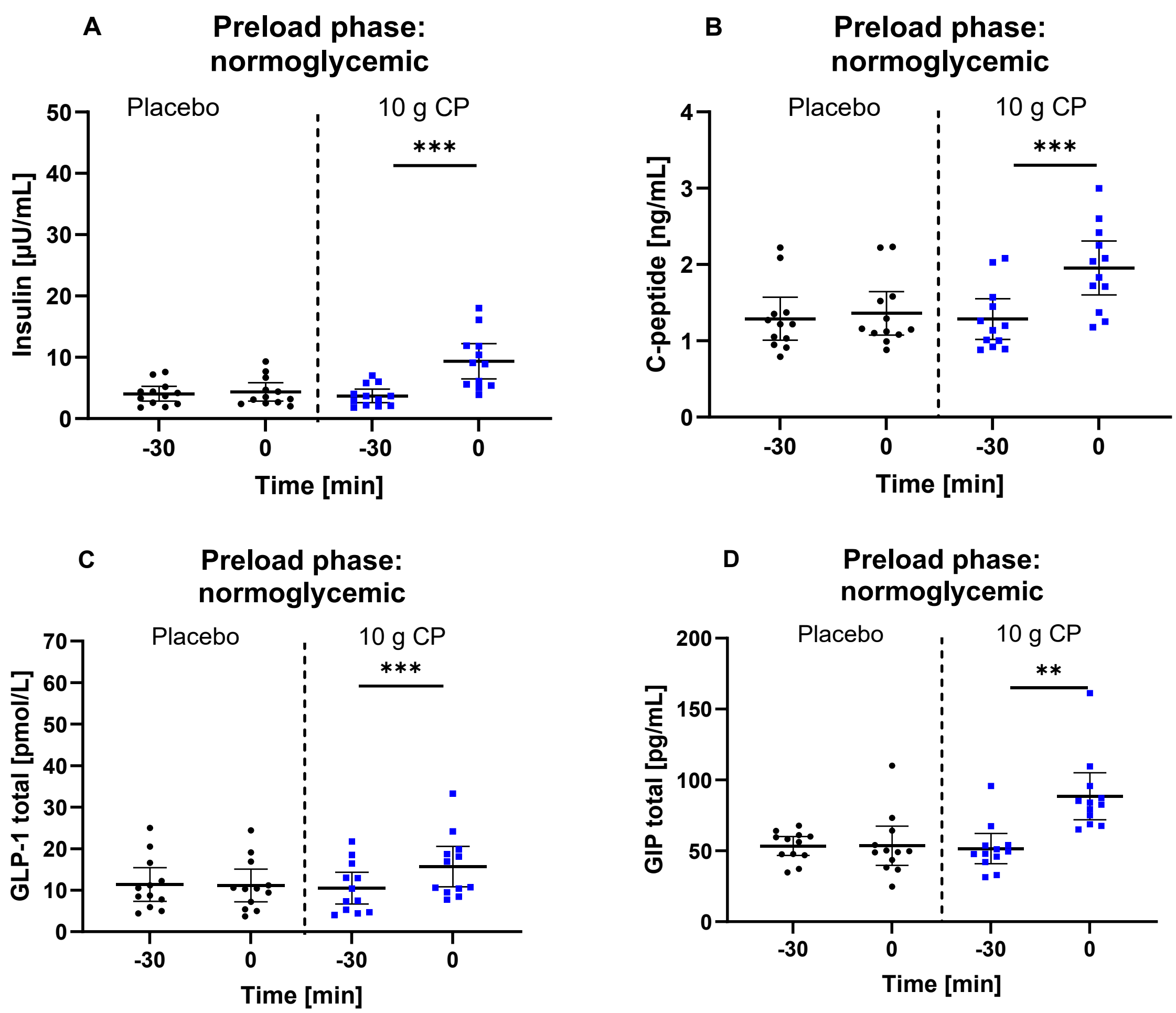

Supplementary Figure S2: Scatter diagram with mean±95% CI of normoglycemic subgroup (n=12) of A) insulin and B) C-peptide C) GLP-1 total and D) GIP total in preload phase with intake of study products placebo or 10 g CP at -30 min; **p<0.01; ***p<0.0001

Supplementary Figure 3

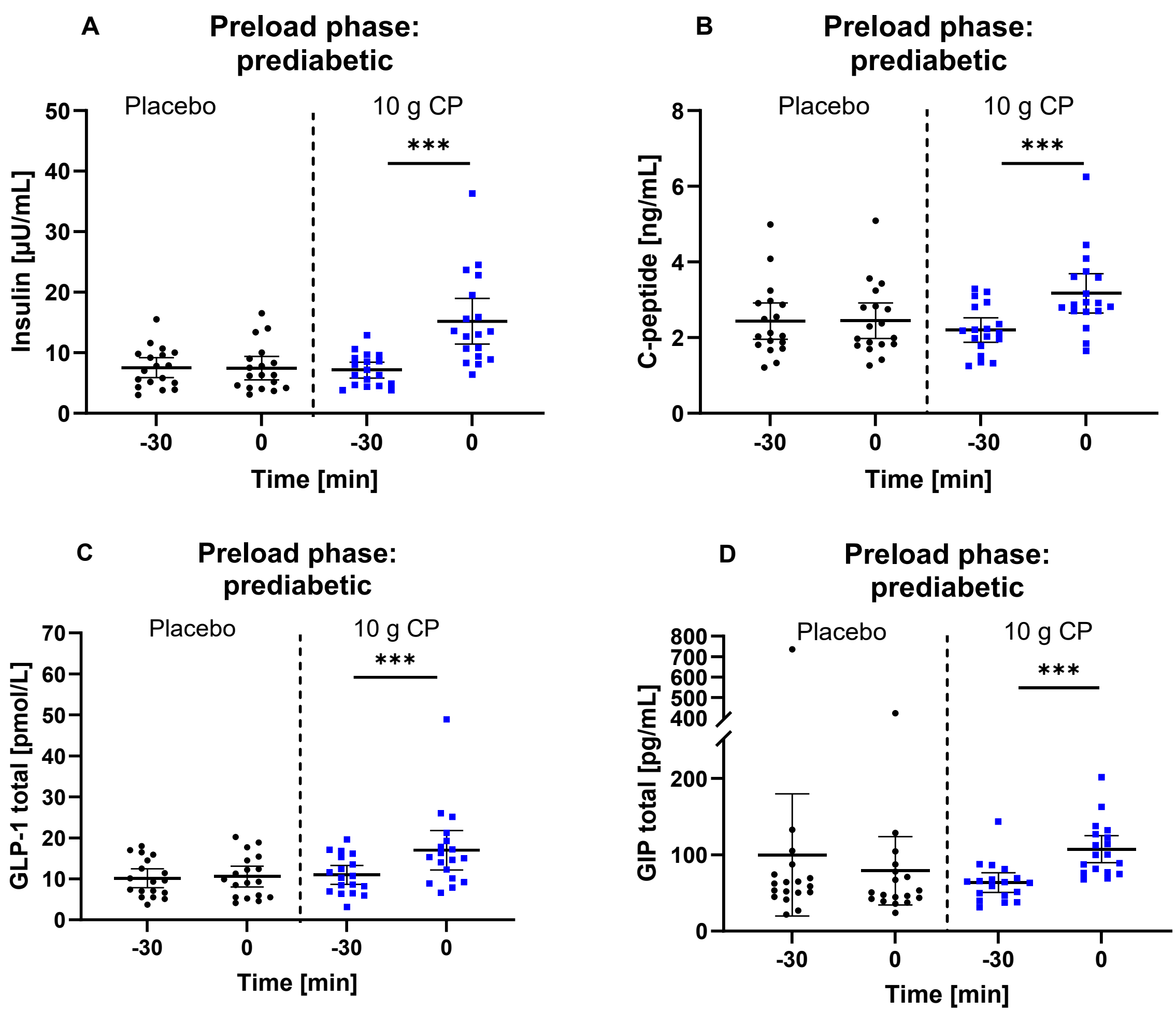

Supplementary Figure S3: Scatter diagram with mean±95% CI of prediabetic subgroup (n=18) of A) insulin, B) C-peptide, C) GLP-1 total and D) GIP total in preload phase with intake of study products placebo or 10 g CP at -30 min; ***p<0.0001

Supplementary Figure 4

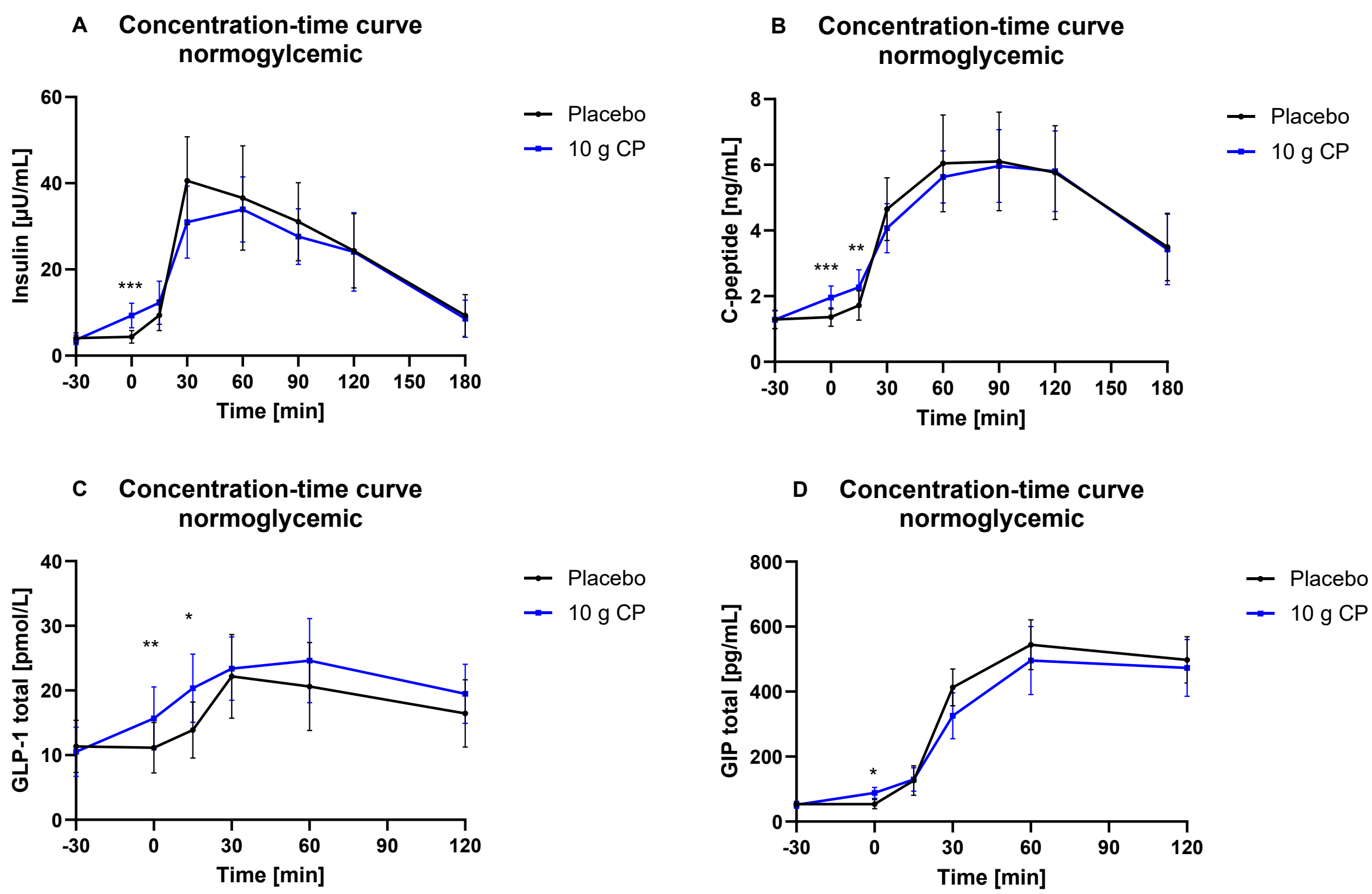

Supplementary Figure S4: Concentration-time curves in normoglycemic subgroup of A) insulin, B) C-peptide, C) GLP-1 total and D) GIP total response after intake of study products (-30 min) and mixed meal (0 min) (mean±95% CI); *p<0.05, **p<0.01; ***p<0.001

Supplementary Figure 5

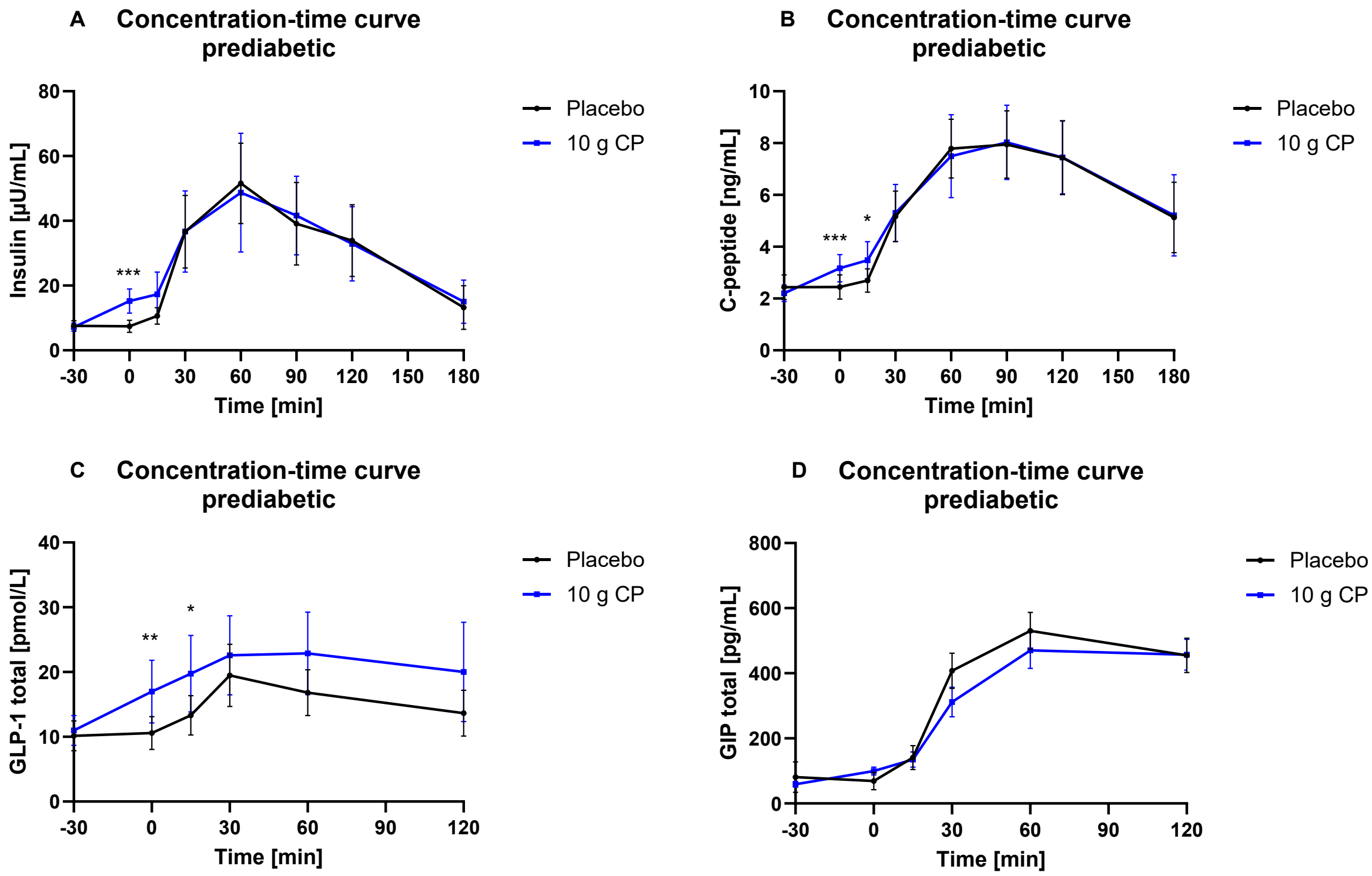

Supplementary Figure S5: Concentration-time curves in prediabetic subgroup of A) insulin, B) C-peptide, C) GLP-1 total and D) GIP total response after intake of study products (-30 min) and mixed meal (0 min) (mean±95% CI); *p<0.05, **p<0.01; ***p<0.001
